# How selves differ within and across cognitive domains: self-prioritisation, self-concept, and psychiatric traits

**DOI:** 10.1186/s40359-022-00870-0

**Published:** 2022-06-30

**Authors:** Kelsey Perrykkad, Jakob Hohwy

**Affiliations:** 1 Cognition and Philosophy Lab, Philosophy Department, School of Philosophical, Historical and International Studies, Monash University, 29 Ancora Imparo Way, Clayton, VIC 3800 Australia; 2 Monash Centre for Consciousness & Contemplative Studies, Monash University, Clayton, Australia

**Keywords:** Self-cognition, Self-concept, Self-prioritisation, Shape-label matching, Psychiatric traits, Autism, Schizophrenia, Borderline personality disorder, Depression, Anxiety

## Abstract

**Background:**

How we build and maintain representations of ourselves involves both explicit features which are consciously accessible on reflection and implicit processes which are not, such as attentional biases. Understanding relations between different ways of measuring self-cognition both within and across such cognitive domains is important for understanding how selves may differ from one another, and whether self-cognition is best understood as largely uni-dimensional or more multi-dimensional. Further, uncovering this structure should inform research around how self-cognition relates to psychiatric and psychological conditions. This study explores the relations between different constructs of self-cognition and how variability within them relates to psychiatric traits.

**Methods:**

Our final dataset includes within-subject (n = 288, general population) measures of explicit self-concept (using both the Self Concept Clarity Scale and Self Concept and Identity Measure), implicit self-prioritisation in a shape-label matching task (for both reaction time and sensitivity) and measurement of traits for five psychiatric conditions (autism, borderline personality disorder, schizophrenia, depression and anxiety). We first test whether self-cognitive measures within and across domains are correlated within individuals. We then test whether these dimensions of self-cognition support a binary distinction between psychiatric conditions that either are or are not characterised in terms of self, or whether they support self-cognition as transdiagnostically predictive of the traits associated with psychiatric conditions. To do this we run a series of planned correlations, regressions, and direct correlation comparison statistics.

**Results:**

Results show that implicit self-prioritisation measures were not correlated with the explicit self-concept measures nor the psychiatric trait measures. In contrast, all the psychiatric traits scores were predicted, to varying degrees, by poorer explicit self-concept quality. Specifically, borderline personality disorder traits were significantly more strongly associated with composite explicit self-concept measures than any of depression, anxiety, or autism traits scores were.

**Conclusions:**

Our results suggest that selves can differ considerably, along different cognitive dimensions. Further, our results show that self-cognition may be a promising feature to include in future dimensional characterisations of psychiatric conditions, but care should be taken to choose relevant self-cognitive domains.

## Background

**Fig. 1:**
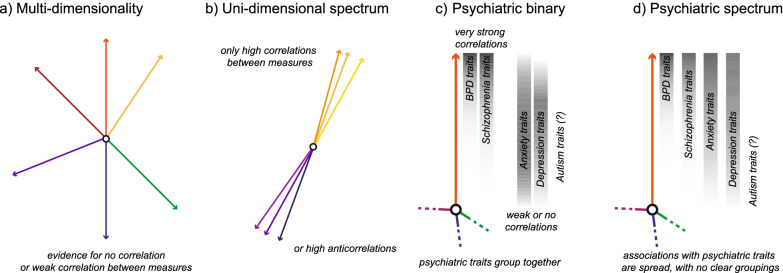
Conceptual Possibilities for the Relations between Self Constructs and Psychiatric Traits. Each coloured arrow depicts one construct of the self (e.g. self-prioritisation, self-concept, bodily self, agency etc.). Panels **a** and **b** show two extremes of the possible relations between these constructs, where all of them are approximately orthogonal (panel **a**) or where they tightly correlate along one axis (panel **b**). In the multi-dimensional version, there are many more ways in which selves can differ, as an individual can fall at different values for each arm independently. Panels **c** and **d** show two ways in which each axis might relate to psychiatric conditions and their traits. In the case where the traits of some conditions covary with a self-cognitive dimension and others don’t, a binary separating psychiatric conditions as related to that self-construct or not is appropriate (panel **c**). If the psychiatric conditions all covary with a given self-construct, but to different degrees, this suggests a spectrum of relation to psychiatric traits for that construct is more appropriate (panel **d**). The latter affords a more nuanced fingerprint of each psychiatric condition as it relates to each self-construct, especially if one integrates this pattern across all arms of panel **a**

Our multifaceted sense of self plays an important role in giving our lives meaning. Differences in the sense of self affect mental well-being [1, 2]. However, the variability of selves remains poorly understood. From a cognitive standpoint, how we build and maintain representations of ourself—our own body, dispositions, name, history etc.—involves both conscious and unconscious resources from across sensory domains and at different levels of the cognitive hierarchy (i.e. integrating information at increasing levels of abstraction). For instance, when and how low-level sensory information is processed can be affected simply by nominally relating otherwise meaningless stimuli to the self [3]. People remember traits that they think they have, and actions that they performed themselves, better than those they attribute to others [4, 5]. One’s self-concept assimilates many years of personal history and changes across the lifespan [6]. There is little research about whether and how many of these self-cognition constructs are related within an individual (cf. [7–11]). For instance, it is unknown whether a person with a more stable and consolidated self-concept is also likely to be a person who has a greater perceptual self-bias, and a greater mnemonic self-bias (Fig. 1b). Or is it rather that each of these self-related constructs is largely independent within an individual (Fig. 1a). Figure 1 depicts the possible conceptual space considered in this paper along two dimensions. If self-cognitive constructs are all tightly correlated, then this greatly limits the degree to which selves can differ (see Fig. 1b). If not, then claims about the nature of ‘the self’ need to specify along which dimension(s) they pertain (see Fig. 1a).

In this paper, we focus on uncovering some of the relations between implicit and explicit features of the self. Constructs that measure implicit processes are defined here as those which affect sensory processing, but are not necessarily consciously accessible to the individual (and are therefore often at a low level of abstraction). Recent evidence from Nijhof et al. [8] shows that the magnitude of two low-level measures of self-prioritisation, namely reduced attentional blink for own name and increased association between arbitrarily-paired self-labels and shapes, were not correlated within individuals. In contrast, Amodeo et al. [7] showed a positive relationship between the magnitude of self-bias in the same shape-label matching task and a visual search for own name task within individuals. This line of previous research measures the relations between constructs within the implicit category.

Self-cognition also involves processes related to the conscious, self-reflective constructs of the self, which are more explicit or apparent to the individual. Previous research has found positive associations between different measures within the category of explicit self-representation, for example, Nowicka et al. [11] found that participants with high self-esteem showed greater neural self-preference when evaluating traits for self-attribution.

There is very little existing research comparing across explicit and implicit self-cognitive dimensions. Krol et al. [10] compared self-reported self-concept clarity and multisensory bodily illusions (rubber hand illusion and body swap illusion) showing that participants with poorer self-concept were more susceptible to these illusions. These findings suggest that a less well-established explicit self-concept may be associated with a less stable sense of the bodily self.

It is important to understand how these self-representational processes both within and across levels of the cognitive hierarchy relate to one another as it may help uncover (or constrain) the cognitive and psychological mechanisms through which we come to have a particular sense of self. Knowing these relationships is beneficial for the development of effective tools for clinical assessment of, and intervention on, the aspects of self-cognition that have the greatest impact on well-being and thriving. The first aim of our study is therefore to investigate relationships between self-cognitive constructs both within and across cognitive levels, spanning the implicit/explicit divide. We do this by comparing implicit self-cognitive measures from the shape-label matching task mentioned above (cf. [3, 7, 8]) and two self-report measures of explicit higher-order self-concept.

In the shape-label matching task, participants must respond to a presented shape and label and decide whether they match a learned pairing. Generally, participants are faster and more discriminant in response to shapes learned as representing oneself as compared to representing others. Specifically, we measure the magnitude of this bias (aka self-prioritisation or self-advantage) in reaction time and sensitivity (d′), and compare these magnitudes within individuals for this implicit self-cognition domain. For the explicit self-concept domain, we use the Self-Concept and Identity Measure (SCIM) [12] and the Self-Concept Clarity Scale (SCCS) [13]. These questionnaires ask individuals to make reflective judgements about the structure of their self-concept which relate to its stability, consistency, and certainty (SCCS), and whether it is consolidated, disturbed, or lacking (SCIM). These tasks are designed to reveal the more conscious and subjectively evaluated self. Like the implicit task, we compare within the explicit self-concept domain by comparing SCIM and SCCS scores within individuals. Further, we compare the shape-label matching task to the explicit self-concept measures. If we find that these measures across the two domains are correlated (or anticorrelated), it would be evidence that self-cognitive constructs across the cognitive hierarchy cluster, as if under a broader uni-dimensional spectrum (Fig. 1b) rather than sitting more orthogonally in a multi-dimensional space (uncorrelated, Fig. 1a). If not, then this would support the model in which there are more dimensions along which individual selves can differ. Of course, these are just two of many more self-related constructs, but determining their relations in detail can meaningfully add to the available evidence for adjudicating between these models of the ways in which selves can differ. The differences between these models have implications for how we understand the relations between self-cognition and other meaningful features of our mental lives. For example, when making sense of reported experiences of depersonalisation or of no-self during meditation, knowing whether the explicit report of such self-experiences relates to sensory processing has implications for how we conceptualise these states and, in more negatively-valenced cases, how we might treat them.

The question of whether and how self constructs cluster around an underlying axis is particularly salient in psychiatric conditions. Indeed, some psychiatric conditions are partially defined in terms of self-cognition, whereas others are not. As defined in the International Statistical Classification of Diseases (ICD-11) [14], conditions defined in terms of self-cognition include borderline personality disorder (BPD) and schizophrenia (see Table 1). These can be distinguished from other psychiatric conditions such as depression and anxiety, which do not involve self-cognition in their ICD or Diagnostic and Statistical Manual (DSM-5) characterisations [14, 15] (see Table 1). This definitional approach offers a binary conception of conditions that do relate to the self vs. those that do not. On such a binary conception, the extent to which selves differ is limited to these two major categories (Fig. 1c). There is increasing debate in the psychiatric field about whether conditions are better defined, like this, in terms of the presence and absence of condition-specific criteria, or by the ways they vary along a transdiagnostic set of biobehavioural dimensions [16]. In this study, we use measures of psychiatric traits in the general population as an early step in determining how to conceive of the relationship between self-representation and the signs and symptoms that are relevant to psychiatric conditions. There is some evidence that issues with self-cognition, such as identity disturbances, are transdiagnostically relevant, including for conditions not defined in terms of the self, such as depression and anxiety [17, 18]. Therefore, we suggest, if the strength of the relations between self constructs and psychiatric traits are distributed along a spectrum, then it would appear self-cognition is relevant in predicting psychiatric traits for a range of conditions, and not just those that are associated with self-representation by their definition (see Fig. 1d).

**Table 1:** Self-disorder classifications and ICD-11

Psychiatric condition	Classification for this study	Relevant ICD-11 description excerpt [14]
Personality disorder: borderline pattern (*Borderline Personality Disorder*)	Characterized by self-disturbances	*“Personality disorder is characterised by problems in functioning of aspects of the self (e.g., identity, self-worth, accuracy of self-view, self-direction), and/or interpersonal dysfunction…” … “The borderline pattern descriptor may be applied to individuals whose pattern of personality disturbance is characterised by a pervasive pattern of instability of interpersonal relationships, self-image… identity disturbance, manifested in markedly and persistently unstable self-image or sense of self;…”*
Schizophrenia	Characterized by self-disturbances	*“Schizophrenia is characterized by disturbances in multiple mental modalties, including… self-experience (e.g., the experience that one’s feelings, impulses, thoughts or behaviour are under the control of an external force)…”*
Depressive disorders (*Depression*)	Not characterized by self-disturbances	*“Depressive disorders are characterised by depressive mood (e.g., sad, irritable, empty) or loss of pleasure accompanied by other cognitive, behavioural, or neurovegetative symptoms that significantly affect the individual’s ability to function.”*
Anxiety	Not characterized by self-disturbances	*“Apprehensiveness or anticipation of future danger or misfortune accompanied by a feeling of worry, distress, or somatic symptoms of tension. The focus of anticipated danger may be internal or external.”*
Autism spectrum disorder (*Autism*)	Not characterized by self-disturbances	*“Autism spectrum disorder is characterised by persistent deficits in the ability to initiate and to sustain reciprocal social interaction and social communication, and by a range of restricted, repetitive, and inflexible patterns of behaviour, interests or activities that are clearly atypical or excessive for the individual’s age and sociocultural context.”*

Previous research, using some of the same measures that will be used in the current study, support the idea that differences in explicit self-cognition are associated with both psychiatric diagnoses and their traits more broadly. Poorer explicit self-concept as measured by SCCS has been established in individuals with schizophrenia [19] and BPD [20]. Lower SCCS scores have also been associated with more depressive symptoms as reported by the Beck Depression Index (BDI) short [21], and BDI-II [22]; and anxiety symptoms as measured by the Beck Anxiety Index (BAI) [22]. Lower SCIM scores have also been associated with BPD, depression [12, 23], and the depression-anxiety scale [2]. Additionally, previous research investigating associations between performance on this shape-label matching task and the psychiatric traits of interest here shows differences depending on mood inductions related to depression and anxiety [24, 25]. Indeed, in one study, sub-clinically anxious individuals did not show the usual significant self-bias at all [26]. If differences in implicit self-bias are related to traits and symptoms of conditions that are defined by self-cognitive features as well as for those that are not, this would suggest that self-cognition is transdiagnostically relevant (as in Fig. 1d). However, for individuals with clinical depression, two previous studies have found no differences from the neurotypical bias in this shape-label matching task [27, 28]. The task has not, to our knowledge, been studied in relation to either schizophrenia or BPD thus far. As such, existing data is inconclusive on this point. Comparing these two categories of psychological traits on both implicit and explicit measures of self-cognition will help arbitrate between a binary or spectrum model of their relations (Fig. 1c or d).

In this study, we focus on traits of Autism Spectrum Condition (autism, ASC) as a key test case for resolving the issue of whether psychiatric traits distribute themselves in a binary or spectrum-like fashion along the dimensions of self-related processing. We investigate how similar or dissimilar autism traits are from traits for those conditions defined in terms of the self and those not so defined. Based on its diagnostic criteria alone, autism would be considered a psychiatric condition that is not characterised by differences in self (see Table 1), but there is growing awareness that autism is in fact associated with differences in many self-representation constructs [29–37]. Making comparisons between autistic traits and schizophrenic traits in particular may be an area where the dimensional approach to psychiatric conditions can be valuable [38, 39]. Research using biological measures such as functional magnetic resonance imaging [40] and genetic markers [41–43] has found shared features between the two conditions, but also key differences. Other work has suggested that the two conditions present as opposites for some cognitive processes [44], including mentalizing [45], and understanding oneself [46]. This positions autism as an informative case study for determining the transdiagnostic utility of measuring explicit and implicit dimensions of self-related processing. By studying traits for these conditions within participants in a general population, we can provide further evidence pertaining to whether self-related processing is a meaningful separator or a shared dimension.

There is evidence to suggest that both low-level attentional and higher-order self constructs show particular differences in autism. One of the early indicators of autism in young children is reduced attention to own name, which has been demonstrated behaviourally and neurally in both autistic children and adults [47–53]. While arbitrary shape-label matching tasks like the one used here generally show no relationship with autism diagnosis or AQ score [8, 54, 55], there is some evidence that autistic participants use different self-cognitive attentional processes despite a similar magnitude of self-bias [55]. Self-concept too, appears to be different in autism. For example, in contrast to neurotypical self-report, autistic participants have claimed that their own self-knowledge is not as accurate as the perception others have of them [56]. Using one of the explicit self-concept questionnaires we chose here, Berna et al. [57] show that higher scores on the autism spectrum quotient (AQ) are associated with a less clear self-concept. So, it is plausible that differences in autistic self-cognition involve implicit attentional mechanisms and explicit conceptual representations.

In this study, we aim to better understand the cognitive structure of the self in order to investigate both along which dimensions selves can differ and how selves differ along each dimension. First, we ask whether self-representations across the cognitive hierarchy are related within individuals; specifically, the quality of relatively low-level attentional self-prioritisation (implicit self-representation) and relatively high-level self-concept (explicit self-representation). Second, we ask whether and in what way psychiatric traits, and autistic traits in particular, vary along these self constructs. To our knowledge, no study has directly statistically compared the strength of these relationships with any self construct between different diagnosed populations, or by directly contrasting their association with different conditions’ traits. This study makes a first step towards directly comparing these relationships transdiagnostically.

If attentional self-prioritisation and self-concept are built on the same domain-general architecture in the general population, and neuronal messages are passed between levels of the cognitive hierarchy relatively seamlessly, we would expect the quality of self-concept as measured by the SCCS and SCIM to be correlated with implicit self-prioritisation measures from the shape-label matching task (Fig. 1b). In contrast, if self-cognition is fractionated across domains, then there is less reason to expect explicit and implicit self measures to be correlated (Fig. 1a).

If psychiatric conditions are distributed in a binary fashion, as traditional diagnostic characterisations would suggest, then the relation between each of the BPD and schizophrenia traits and the self-constructs should be significantly stronger than each of the anxiety and depression traits and the self-constructs, and our test case of autistic traits should fall into one of these two groups (Fig. 1c). If, in contrast, the transdiagnostic picture is more adequate, then we should see the correlations with traits for these different conditions distributed across a spectrum for each dimension (Fig. 1d). Notably, this may differ across self-domains, if self-cognitive constructs are not found to cluster within an individual (Fig. 1a as opposed to Fig. 1b). The resulting combination, from Fig. 1, of 1a versus 1b and 1c versus 1d will be informative for understanding if and how selves can differ from one another across and within self constructs.

## Methods

This study was approved by Monash University Human Research Ethics Committee (Project Number 23583) and was conducted in accordance with the relevant guidelines and regulations. All participants gave informed consent upon commencing the protocol.

### Participants

A total of 328 participants successfully completed the study posted on Amazon Mechanical Turk using the Cloud Research platform (formerly TurkPrime [58]), with an overall completion rate of 70% (30% accepted but did not complete the posting) and a bounce rate of 9% (decided not to complete the study after viewing the description). Data was collected between June 26 and July 30, 2020. Participants were paid $9 USD for completing the task, which took an average of 64 min to complete (including consent process and self-timed breaks to a maximum of 180 min total task duration). A total of 40 participants were excluded for the following reasons: uncorrected issue with vision (n = 2), previous head injury which resulted in temporary unconsciousness (n = 2), more than one missed manipulation check (> 10%, n = 4) [59], performance on the self-prioritisation task which was more than two standard deviations below mean (i.e., < 31% overall accuracy, n = 9), more than 50% of self-prioritisation task trials removed or an overall mean greater than two standard deviations above the average for reaction time on the shape-label matching task (n = 31). Participant demographic information for the final dataset from 288 participants is available in Table 2.

**Table 2:** General demographic information

Demographic	Category	N	% (Total N = 288)
Gender	Male	155	54.2
	Female	128	44.4
	Other	5	1.7
Age	18–24	46	16.0
	25–31	79	27.4
	32–38	95	33.0
	39–45	55	19.1
	46–50	25	8.7
Country of residence	USA	283	98.3
	Canada	5	1.7
First language	English	276	95.8
	Other—fluent in English	12	4.2
Highest completed education	Highschool or equivalent including Vocational Training	71	24.7
	Bachelors, Honours or Associate Degree	164	56.9
	Masters or Doctorate	53	18.4
Employment status	Unemployed or not working	36	12.5
	Student or intern	19	6.6
	Employed	233	80.9
Official diagnoses	Autism Spectrum Disorder/Autism/Autistic Disorder/Aspergers’ Syndrome/Pervasive Developmental Disorder-Not Otherwise Specified (PDD-NOS)	3	1.0
	Borderline Personality Disorder	2	0.7
	Schizophrenia	0	0
	Depression	43	14.9
	Anxiety	49	17.0
	Attention-Deficit/Hyperactivity Disorder	3	1.0
	Bipolar Disorder	2	0.7
	Obsessive Compulsive Disorder	1	0.3
	Posttraumatic Stress Disorder	1	0.3
	None	224	77.8
Reported comorbidity in diagnoses of interest	Autism Spectrum Disorder/Autism/Autistic Disorder/Aspergers’ Syndrome/PDD-NOS & Depression & Anxiety	1	0.3
	Autism Spectrum Disorder/Autism/Autistic Disorder/Aspergers’ Syndrome/PDD-NOS & Depression	2	0.7
	Borderline Personality Disorder & Depression & Anxiety	2	0.7
	Depression & Anxiety	31	10.8

### Procedure

Psychiatric traits for the five conditions were measured using the Autism-Spectrum Quotient (AQ) [60], Borderline Personality Questionnaire (BPQ) [61], Schizotypal Personality Questionnaire (SPQ) [62], Beck Anxiety Inventory (BAI) [63] and Beck Depression Inventory (BDI) [64]. Explicit self-concept was measured by two questionnaires, the Self-Concept and Identity Measure (SCIM) [12], and the Self-Concept Clarity Scale (SCCS) [13]. Implicit self-prioritisation was measured using a label-shape matching task [3]. The demographic information, AQ, BPQ and SPQ were completed in that order before the self-prioritisation task. The SCCS, SCIM, BAI and BDI were completed following the task. This order did not change across participants.

### Psychiatric trait survey measures

#### Autism-spectrum quotient (AQ)

The AQ is a 50-item questionnaire measuring autistic traits in the general population. Items are rated on a four-point Likert scale and scored on a two-point scale (all responses of the same valence are collapsed for scoring). The questionnaire covers the autistic features related to social skills, attention switching, attention to detail, communication, and imagination [60]. While we acknowledge that some uses of this scale in making conclusions about core features of autism have recently been criticised [65, 66], it remains the most widely used for measuring autistic traits in a general population. Cronbach’s alpha for the internal consistency of AQ in our sample was 0.80 (good).

#### Borderline Personality Questionnaire (BPQ)

The BPQ is an 80-item questionnaire measuring borderline personality traits as defined by the DSM-IV criteria. Items cover features of borderline personality disorder including impulsivity, affective instability, abandonment, relationships, self-image, suicide or self-mutilation, emptiness, intense anger and quasi-psychotic states [61]. Participant responses consist of true/false judgements. Cronbach’s alpha for the internal consistency of the sum score for BPQ in our sample was 0.94 (excellent).

#### Schizotypal Personality Questionnaire (SPQ)

The SPQ is a 74-item questionnaire measuring schizotypy based on the DSM-III-R criteria for Schizotypal Personality Disorder. Features of schizophrenia which are covered by the questionnaire include ideas of reference, social anxiety, odd beliefs and magical thinking, unusual perceptual experiences, eccentric or odd behaviour and appearance, no close friends, odd speech, constricted affect and suspiciousness or paranoid ideation [62]. Participants respond to each item with Yes or No. Cronbach’s alpha for the internal consistency of the sum score for SPQ in our sample was 0.95 (excellent).

#### Beck Anxiety Inventory (BAI)

The BAI is a 21-item questionnaire measuring the recent presence and severity of anxiety symptoms. Items cover symptoms such as fear, inability to relax, numbness, sweating, dizziness, and heart-racing [63]. Participants report how often they have been bothered by each symptom of anxiety in the last month on a four-point likert scale (“Not at all”, “Mildly…”, “Moderately…”, “Severely…”). Cronbach’s alpha for the internal consistency of BAI in our sample was 0.92 (excellent).

#### Beck Depression Inventory (BDI)

The BDI is a 21-item questionnaire measuring the recent presence and severity of depressive symptoms. The items address features including sadness, pessimism about the future, sense of failure, lack of satisfaction/pleasure, guilt, sense of punishment, self-hatred, self-blame, suicidal thoughts, crying, irritability, social interest, indecision, body image, work, sleep disturbance, fatigue, appetite, weight loss, health concerns and libido [64]. Participants choose one of four options for each item with increasing severity of descriptions for depression symptoms with reference to the last few weeks. Cronbach’s alpha for the internal consistency of BDI in our sample was 0.94 (excellent).

### Explicit self-concept survey measures

#### Self-Concept and Identity Measure (SCIM)

The SCIM is a 27-item questionnaire measuring dimensions of healthy and disturbed identity as understood as a core component of personality pathology in the DSM-5. Items are rated on a seven-point Likert scale (“Strongly Agree”… “Neither agree nor disagree”… “Strongly Disagree”). Higher scores are indicative of “greater identity disturbance” [12]. Cronbach’s alpha for the internal consistency of total scores on the SCIM in our sample was 0.93 (excellent). In its initial development, the SCIM was found to have a three-factor structure, and can be broken down into measures of Disturbed Identity (11-items), Consolidated Identity (11-items) and Lack of Identity (6-items). A confirmatory factor analysis of this structure did not yield strong evidence for this factor structure in our sample (CFI = 0.81, RMSEA = 0.10), however, Cronbach’s alpha was good–excellent within each factor (Disturbed Identity: α = 0.88, Consolidated Identity: α = 0.85, Lack of Identity: α = 0.91). Only the full score was used in subsequent analysis.

#### Self-Concept Clarity Scale (SCCS)

The SCCS is a 12-item questionnaire measuring self-concept structure. Each item is rated on a five-point Likert scale (“Strongly Agree”… “Neutral”… “Strongly Disagree”). Higher scores are related to increased clarity of self-concept, including temporal stability, certainty and perceived internal consistency of beliefs about oneself. Low scores were independently associated with chronic self-analysis, lower internal state awareness, and ruminative self-focused attention [13]. Cronbach’s alpha for the internal consistency of SCCS in our sample was 0.87 (good).

It should be highlighted that high scores on the SCCS and low scores on the SCIM relate to better quality self-concept, and thus are expected to be anti-correlated.

### Self-prioritisation task

Implicit, perceptual self-prioritisation was measured using a shape-label matching task [3]. The self-prioritisation task was run using Inquisit Web ([67], Retrieved from: https://www.millisecond.com). In this task, participants were presented with three pairings of a shape and label. The labels used were “self”, “friend” and “stranger” which were paired with a circle, triangle and square (mappings counterbalanced across participants). In each trial, participants are asked whether a briefly presented (100 ms) shape and label matched. Trials falling into the six possible pairings (self-match, self-mismatch, friend-match, friend-mismatch, stranger-match, stranger-mismatch) were equally probable and randomly ordered within three blocks of 120 trials for a total of 360. All stimuli were white and presented on a grey background following a 500 ms central fixation cross. After each trial, participants received feedback as to whether they were correct and further percent accurate feedback was given at the end of each block. The response was speeded, and if participants were too slow (random window of 800–1200 ms), a warning appeared following that trial.

From performance on this task, two measures of implicit self-representation were computed. The first measure was based on reaction time, which is the time in milliseconds from the offset of the stimuli until the response. The second measure is based on signal detection sensitivity, or d′. This measure combined matching and nonmatching shape trials to give an unbiased measure of the separation between distributions between signal and noise in units of standard deviation for the signal distribution. For our analysis, we focus on self-advantage measures for both d′ (self-shape trials minus the average of friend and stranger trials) and reaction time (congruent self-shape trials minus the average of congruent friend and congruent stranger trials). This gives us a measure of self-bias, called *self-advantage*, in both average reaction time and sensitivity for each individual. Note that faster reaction times in the self condition would give a more negative self-advantage score, so greater self-bias is evidenced by larger d′ self-advantage but numerically smaller reaction-time self-advantage. An alternative dataset is available for interested readers using all trials for the calculation of the reaction time self-advantage measure (https://doi.org/10.26180/14214464).

### Statistical analysis

Where possible, statistical analyses are reported in both traditional null-hypothesis significance testing (NHST) and Bayesian form using JASP v0.9.0.1 [68] through Jamovi v1.1.9.0 [69], and R v3.6.3 software [70]. For Bayes factor interpretation, ‘anecdotal’ evidence is used to describe Bayes factors greater than 1 and less than 3, ‘substantial’ is used for Bayes factors greater than 3, ‘strong’ for greater than 10, ‘very strong’ for greater than 30, and ‘extreme’ for Bayes factors greater than 100, following Jeffreys [71]. BF_10_ > 1 indicates evidence in favour of the alternative hypothesis (H1) over the null hypothesis (H0), where BF_01_ > 1 indicates evidence for the null, which can also be inferred by increasingly small fractions of BF_10_ < 1.

#### Explicit and implicit self-representation

To investigate the relationship between implicit and explicit self-representation and within each domain, we conduct Pearson’s correlations between the two explicit questionnaires and the two self-advantage measures. For the matching Bayesian correlations we use a stretched beta prior of width one. The NHST correlations are Bonferroni corrected for the six pairwise comparisons both within and between explicit and implicit self measures. Post-hoc power calculations for the correlations between self-cognitive measures (two-tailed correlations with Bonferroni-corrected significance threshold alpha and final sample size of 288, G*Power version 3.1.9.7) indicated any true correlation of 0.2 or greater would be detected with at least 0.95 probability.

#### Psychiatric traits and self measures

We partition the psychiatric traits analyses into three steps. Through these analyses, we quantify and compare the relationships between psychiatric traits and the self measures and particularly whether autism is more similar to conditions characterised by self-cognition or to those that are not (and whether this binary conception holds at all). The first step is to look at the relationships between the traits scores and the self measures in their simplest form—using pairwise correlations as we did for the first question. Significance of the NHST correlations are Bonferroni corrected for the 20 pairwise comparisons. Post-hoc power calculations for these correlations (two-tailed correlations with Bonferroni-corrected significance threshold alpha and final sample size of 288, G*Power version 3.1.9.7) indicated any true correlation of 0.27 or greater would be detected with at least 0.95 probability.

The second step compares the strength of relationships between self measures and psychiatric traits indirectly, by quantifying variance in each trait score which is explained by the self measures. To do this, we perform a multiple linear regression with each psychiatric trait score as the predicted variable and each of the four self measures as predictors. Bayesian regressions are run with a JZS prior with r scale of 0.354, uniform model priors and use the BAS sampling method, all of which are default in Jamovi. Comparing the R-squared values across these models further illuminates whether the cluster of self measures as a whole best predicted the traits for conditions defined by self differences.

The final step is to directly statistically test whether the self measures distinguish between conditions that are defined by self differences and those that are not. There was no known Bayesian method for this part of the analysis. To minimise the number of comparisons, we perform principal component analysis (PCA) with varimax rotation on the collection of self measures (total scores for each measure as above) as a dimensionality-reduction technique. The top components are selected at an eigenvalue threshold of one. From the loadings variables based on each selected component are created for each participant for further analysis using weighted sum scores [72]. The cocor toolbox [73] is used to directly statistically compare correlations between the psychiatric trait scores and each of the components defined by the PCA analysis, accounting for the dependent (same participants) and overlapping (a shared variable in each comparison) features of the data using ten NSHT methods. As this only allows for pairwise comparisons of correlations, the significance threshold for this family of results was Bonferroni corrected for eight comparisons. Using the same statistical technique, we further test whether the strength of the relationship between AQ and the self measures is significantly different from the other conditions. The significance threshold for this family of results is Bonferroni corrected for eight comparisons. In the vast majority of cases for our data all ten methods agree, and so we report only Pearson and Filon’s z statistic in the reported results. Rare disagreements between methods are also noted, and the more conservative outcome favoured in our interpretation.

## Results

Descriptive statistics for all measures are available in Table 3. Data used for statistical analysis presented below is freely available on Figshare (https://doi.org/10.26180/14214464).

**Table 3:** Descriptive statistics summary

Questionnaire	Mean	Range	1st Qu.	3rd Qu.
Autism-Spectrum Quotient	21.3	4:38	16.0	26.0
Borderline Personality Questionnaire	19.3	0:64	6.0	28.0
Schizotypal Personality Questionnaire	21.4	0:74	10.0	30.3
Beck Depression Inventory	9.6	0:45	2.0	15.0
Beck Anxiety Inventory	8.2	0:40	2.0	12.0
Self-Concept Clarity Scale	43.5	15:60	35.0	52.0
Self-Concept and Identity Measure	68.2	27:145	50.8	81.3
Self-Prioritisation Task *(Overall Accuracy—%)*	76.1	41.7:97.2	66.3	86.7
Self-Prioritisation Task *(d′ Self-Advantage)*	0.76	− 0.81:3.1	0.40	1.1
Self-Prioritisation Task *(Reaction Time (ms) Self-Advantage)*	− 170.4	− 288.2: − 37.4	− 193.7	− 145.45

For the shape-label matching task, while not the focus of the study here, we replicate Sui et al. [3] insofar as d′ is greater for self than friend which is greater than stranger stimuli (F(2,524) = 303.78, *p*_greenhouse-geisser_ < 0.001), and for congruent trials, participants respond faster (F(2,478) = 430.79, *p*_greenhouse-geisser_ < 0.001) and more accurately (F(2,532) = 295.02, *p*_greenhouse-geisser_ < 0.001) for self than others and for friend than stranger.

### Explicit and implicit self-representation

**Fig. 2:**
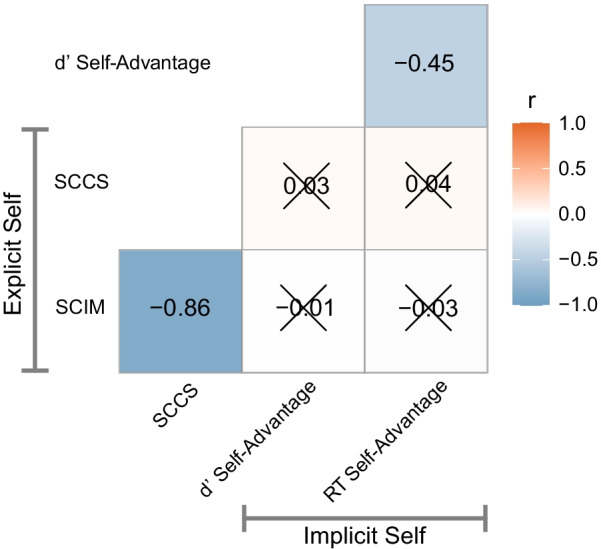
Correlation matrix self measures. Stronger negative correlations are given in an increasingly darker blue shade, and stronger positive correlations in increasingly darker orange. Low SCIM and high SCCS scores indicate better quality self-concept. Similarly, high d' Self-Advantage score (greater sensitivity for self vs. others) and low RT Self-Advantage score (faster reaction time for self vs. others) indicate greater self-bias. Non-significant Bonferroni corrected (six comparisons) Pearson correlations with Bayesian evidence for the null hypothesis are indicated by an X

To investigate the relationship between the explicit self-concept survey scores and the implicit self-prioritisation measures obtained from the task, we performed pairwise correlations. This analysis showed significant relationships within the explicit and implicit measures, but not across (Fig. 2). The explicit SCIM and SCCS scores were strongly negatively correlated (r =  − 0.86, *p* < 0.001, BF_10_ > 100) as expected, given they intend to measure very similar constructs but high scores have opposite meanings. The implicit self measures, d′ self-advantage and reaction time self-advantage, were moderately negatively correlated (r = − 0.45, *p* < 0.001, BF_10_ > 100). This suggests that participants who had a greater difference in sensitivity to self (vs other) also had larger difference in reaction time advantage to self (vs other). All four contrasts between implicit and explicit measures were non-significant by NHST statistics. Bayesian Pearson correlations show that there is evidence for no relationship between explicit and implicit measures (d′ self-advantage and SCCS, BF_10_ = 0.083 (strong); d′ self-advantage and SCIM, BF_10_ = 0.075 (strong); reaction time self-advantage and SCCS, BF_10_ = 0.09 (very strong); reaction time self-advantage and SCIM, BF_10_ = 0.08 (very strong)). This shows that it is not merely that there is a lack of evidence for a relationship between our implicit and explicit self measures, but our data provides evidence against such a relationship.

### Psychiatric traits and self measures

#### Simple correlations

**Fig. 3:**
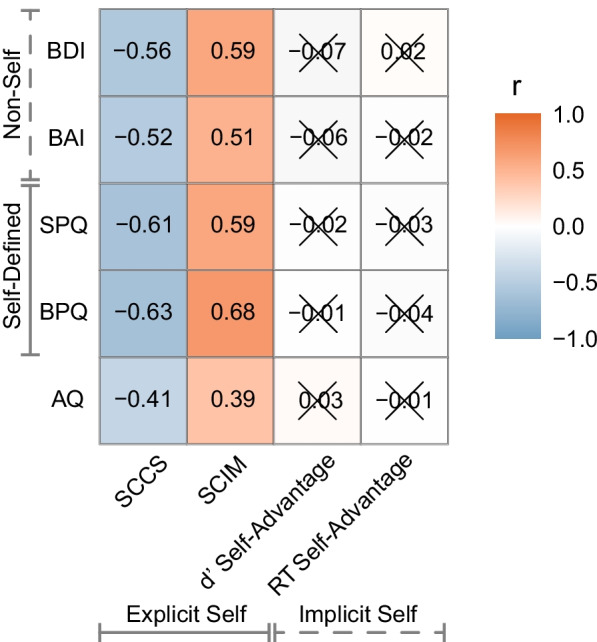
Correlation matrix psychiatric traits and self measures. Psychiatric trait measures are on the y-axis, and self measures along the x-axis. Stronger negative correlations are given in an increasingly darker blue shade, and stronger positive correlations in increasingly darker orange. Non-significant Bonferroni corrected (20 comparisons) Pearson correlations with Bayesian evidence for the null hypothesis are indicated by an X

Our next aim was to probe the strength of the relationship between traits for the psychiatric conditions and self measures. The initial analysis showed that all of the psychiatric trait measures were significantly correlated with the explicit self-concept measures (Fig. 3). Higher psychiatric traits in general are associated with poorer explicit self-concept as measured by both the SCCS and SCIM. As would be expected by their classifications, the conditions not characterised by self have a numerically weaker relationship with the explicit measures than between the self-characterised conditions and SCCS (BAI: r = − 0.52, *p* < 0.001, BF_10_ > 100; BDI: r = − 0.56, *p* < 0.001, BF_10_ > 100; BPQ: r = − 0.63, *p* < 0.001, BF_10_ > 100; SPQ: r = − 0.61, *p* < 0.001, BF_10_ > 100) and SCIM (BAI: r = 0.51, *p* < 0.001, BF_10_ > 100; BDI: r = 0.59, *p* < 0.001, BF_10_ > 100; BPQ: r = 0.68, *p* < 0.001, BF_10_ > 100; SPQ: r = 0.59, *p* < 0.001, BF_10_ < 100). Further, the correlations between AQ and SCCS (r = − 0.41, *p* < 0.001, BF_10_ > 100) and SCIM (r = 0.39, *p* < 0.001, BF_10_ > 100) are numerically weaker than even those for the non-self-conditions.

By traditional NHST correlations, neither of the implicit self measures were correlated with any of the trait measures. There was very strong evidence against a correlation between reaction time self-advantage and any of the psychiatric trait measures (ranging from BF_10_ = 0.076 (AQ) to BF_10_ = 0.093 (BPQ)). There was strong evidence against a correlation between d′ self-advantage and AQ (BF_10_ = 0.083), BPQ (BF_10_ = 0.076) and SPQ (BF_10_ = 0.078), and moderate evidence against a correlation between d′ self-advantage and BAI (BF_10_ = 0.13) and BDI (BF_10_ = 0.14).

#### Regression models

The next step was to compare how well the self measures predicted variance in the trait scores. All of the tested regression models were significant (with extreme evidence for H_1_), demonstrating that at least some of the self measures account for some of the variance in all measured psychiatric trait scales. This is consistent by the conceptual picture in Fig. 1d. Results show that the self measures explain the most variance for BPQ with 47% variance explained, followed by SPQ which is similar to BDI, followed by BAI and lastly, AQ, which has only 17% variance explained. One or both explicit self measures is a significant predictor in all models, consistent with the correlation results above. The implicit self measures did not contribute to the winning regression models. A summary including significant predictors for each model can be found in Table 4.

**Table 4:** Summary of multiple linear regressions

Trait	NHST						Bayesian	
	Adjusted R-squared	F-statistic (4,283)	*p* value	Significant Predictors	t-value	*p* value	BF_10_ winning model	P(M|data)
AQ	0.17	14.9	< 0.001	Intercept	5.23	< 0.001	> 100	0.52
				SCCS	− 2.66	0.0084		
BAI	0.29	28.7	< 0.001	Intercept	2.19	0.029	> 100	0.42
				SCCS	− 3.15	0.0018		
				SCIM	2.36	0.019		
BDI	0.36	40.4	< 0.001	SCIM	4.79	< 0.001	> 100	0.51
SPQ	0.39	44.9	< 0.001	Intercept	3.44	< 0.001	> 100	0.69
				SCCS	− 4.00	< 0.001		
				SCIM	2.94	0.0035		
BPQ	0.47	62.4	< 0.001	SCCS*	− 2.19	0.030	> 100	0.45
				SCIM	5.94	< 0.001		

Significant predictors in NHST regressions matched winning Bayesian model in all cases except for variable marked with * indicating that it was not present in Bayesian winning model. P(M|data) reports the posterior probability of the winning model given the data

#### Direct statistical comparison of correlations

Numerically comparing Pearson’s r from the simple correlations and adjusted r-squared from the regression models suggest that the self measures were least related to AQ score, most related to the self-defined conditions, with the non-self-conditions falling in between. This final part of the analysis directly compares the strength of relationship between the self measures and the traits scores. Following the PCA, the top two components were selected with a cumulative variance of 82.88%. Component loadings are available in Table 5. While PCA components are not interpretable in themselves, these two components neatly map onto our distinction between explicit (Component 1) and implicit (Component 2) self measures. From these loadings, the variables *C1:Explicit* and *C2:Implicit* were created for each participant for further analysis. It should be noted that Multiple Linear Regression models as above using these components as predictors yielded comparable results to those in Table 4.

**Table 5:** PCA analysis details for self-measure dimensionality reduction

Variable	Component 1	Component 2
	*Loadings*	
SCCS	0.966	
SCIM	− 0.965	
d′ self-advantage		0.852
RT self-advantage		− 0.850
	*Eigenvalue*	
	1.87	1.45
	*% of variance*	
	46.69	36.19

To determine whether the correlations between SPQ and BPQ and the self measures were in fact stronger than those with BDI and BAI, we compared correlations within and between our self-defined and non-self-defined categories as reported in Table 6. In summary, C1:Explicit successfully distinguishes between BPQ and both the non-self-defined conditions, but SPQ shows no statistical difference in the relationships with the non-self-defined conditions across all measures. Comparisons between relationships with C2:Implicit are not significant, but neither are any of the first order correlations (Fig. 3). In all cases, the ten methods of comparison used agreed, except when comparing the relationship with C1:Explicit between SPQ and BAI (20% of the methods suggested the null hypothesis should be rejected).

**Table 6:** Comparing correlations between self-defined and non-self-defined psychiatric traits with simplified self measures

	Self-defined conditions							
	BPQ				SPQ			
	C1:Explicit		C2:Implicit		C1:Explicit		C2:Implicit	
	*z*	*p*	*z*	*p*	*z*	*p*	*z*	*p*
**Non-self-defined conditions**								
BDI	− 2.7915	0.0052	0.5180	0.6045	− 0.4312	0.6663	− 0.5489	0.5831
	*		X		X		X	
BAI	− 4.0888	< 0.00001	0.8179	0.4134	− 2.1207	0.0339	− 0.1769	0.8596
	***		X		X^#^		X	

Pearson and Filon’s z: *** = *p* < 0.0001, ** = *p* < 0.0005, * = *p* < 0.00625, X = *p* > 0.00625, # = some disagreement on null hypothesis rejection between statistical methods

Significance threshold α = 0.05/8 = 0.00625

To determine where autism should be placed, we specifically compared the correlations of AQ with the derived components to those with the other conditions (see Table 7). Only in comparing the relationship between AQ and BAI and C1:Explicit did any of the methods reported by the toolbox disagree (20% of the methods indicate that the null hypothesis should be rejected). In summary, AQ has a weaker correlation with C1:Explicit than BPQ, SPQ and BDI do, but (conservatively) the strength of the correlation is not significantly different from that with BAI. Again, the correlation between AQ and C2: Implicit is not significantly different to that of the other trait measures (none of which were significant to begin with, see Fig. 3).

**Table 7:** Comparing correlations between AQ and simplified self measures with other trait measures and simplified self measures

AQ	Compared to							
Correlated with	BPQ		SPQ		BDI		BAI	
	*z*	*p*	*z*	*p*	*z*	*p*	*z*	*p*
C1: Explicit	5.65	< 0.00001	4.63	< 0.00001	3.59	0.0003	2.08	0.0380
	***		***		**		X^#^	
C2: Implicit	− 1.2283	0.2193	− 0.4699	0.6385	− 0.8301	0.4065	− 0.5026	0.6152
	X		X		X		X	

Pearson and Filon’s z: *** = *p* < 0.0001, ** = *p* < 0.0005, * = *p* < 0.00625, X = *p* > 0.00625, # = some disagreement on null hypothesis rejection between statistical methods

Significance threshold α = 0.05/8 = 0.00625

## Discussion

For this study, we used self-report and task data indexing the quality of implicit and explicit self-representations and psychiatric traits scores for conditions characterised in terms of self-cognitive features and those that are not. Participants completed two self-concept surveys, five psychiatric traits surveys and completed a shape-label matching task based on Sui et al. [3]. We did so to better understand whether self-constructs across the cognitive hierarchy and the implicit/explicit divide form a unidimensional or multidimensional structure. Our results show significant correlations between the variables within these categories, and evidence for no relationships across these categories. This suggests a dissociation between explicit self-concept and implicit self-prioritisation within an individual (conceived as Fig. 1a). We were also interested in the structure within each dimension, in particular as these self-constructs relate to psychiatric traits. We find evidence for no relationship between psychiatric traits and implicit measures of self-advantage from the shape-label matching task. The explicit measures however, do correlate with psychiatric traits. We find that some psychiatric conditions (specifically borderline personality disorder) are particularly strongly associated with explicit self-concept, but that all of the studied psychiatric conditions were significantly related to explicit measures of self-concept, albeit to varying degrees. Unexpectedly, we find that autism traits fall below most (if not all) of the other psychiatric traits in terms of their relatedness to explicit self-concept.

### Is there correlation across implicit and explicit self-cognition?

Based on the results presented here, we suggest the structure of self-cognition is multidimensional (i.e., more similar to Fig. 1a than Fig. 1b). Bayesian analysis of our data showed anecdotal to strong evidence that there is no relationship between explicit self-concept, as measured by the SCCS and SCIM, and implicit self-prioritisation, as measured by d′ and reaction time self-advantage from the shape-label matching task. This is consistent with findings of dissociation in self-cognitive measures from Nijhof et al. [8].

Of course, conclusions that can be drawn from this data are limited to the specific cognitive domains studied. Self-cognition has numerous facets that have not been incorporated in the current study, including the bodily self, self in action, memory for self, self-recognition and self-related language use [29]. As we reported earlier, studies such as Krol et al. [10] have found intra-individual relationships between self-cognitive domains. While it is still plausible that low-level attentional mechanisms which typically lead to a prioritisation of the self impact on downstream integrated self-representations at the explicit level, these data suggest that it is not always an easy line to draw from one domain to the other. This may be especially true when comparing very low and very high levels, as we ostensibly did here, between which there are many intervening factors.

### Do these self measures support a binary distinction between psychiatric conditions that are and are not characterised by self-cognition?

While our analysis indicated that BPQ was more strongly associated with explicit self-concept than the conditions not defined in terms of self, it is important to highlight that our analysis did not show any significant differences between SPQ and BAI or BDI on our self measures. It may be relevant to note here that schizophrenia, while defined as having self-features in the ICD-11 [14], is not defined by self-features in the DSM-5, despite involving symptoms which often relate to the self in presentation (for example, ‘delusions’ often involve delusions of control as in the ICD-11 classification, see Table 1) [15]. Borderline personality disorder, on the other hand, involves identity disturbances in both the ICD-11 and DSM-5 criteria, and our results show that its traits are significantly more strongly related to our explicit self measures than traits for either depression or anxiety. This makes it a priori the more prototypical self-characterised condition in our study, supported by our findings that schizophrenia traits seem to sit between borderline personality disorder traits and traits for the other conditions not characterised by the self in the ICD.

Our results, however, suggest it is best to reject the assumption that there is a clear distinction between traits of psychiatric conditions characterised by their relationship with self-cognition and those that are not at the outset. It may be more appropriate to conceive of a transdiagnostic multi-axial spectrum of self-cognition (Fig. 1a & d). This is because self-cognitive measures correlated with and predicted traits for *all* of the studied conditions. Along such a spectrum, high borderline personality disorder traits appear to fall to the furthest extreme of the self-concept dimension. Autism traits appear at the other end of the spectrum, with only 17% variance explained by the self measures; a quantifiably weaker relationship than (almost) all the other conditions studied here. Further, only SCCS scores significantly contributed to this regression (for most other conditions both explicit self measures contributed to the prediction in NHST models). There is still conceptual room below the autism traits correlation on this axis—for conditions that have even weaker or no correlation with self-cognitive measures. We did find a significant association between explicit self-cognition and autism in our dataset, and thus, it would be hasty to dismiss the importance of self-cognition for understanding autism outright.

Our data also suggests that the explicit measures used here are a better candidate for a trans-diagnostic dimension than are responses to the shape-label matching task. This is borne out by the lack of significant correlations between the implicit self measures and any of the psychiatric traits, nor their appearance in the regression models predicting psychiatric traits, and the absence of implicit measures in contributing to discriminability of self-defined conditions from non-self-defined conditions. In contrast, both explicit self measures are correlated with all psychiatric trait measures, at least one of them significantly contributes to the regression model for each psychiatric trait score, and the combination of explicit measures successfully distinguishes BPQ from both BDI and BAI. Qua explicit, these constructs are the kind of thing that is acknowledged and reportable by the individual, and so it is perhaps unsurprising that the traits of the conditions identified by clinicians through interactions would align with these explicit constructs. This is not to say that implicit measures cannot be clinically relevant, just that distinctions which are based on years of clinical observations (as diagnoses are) are likely to be reflected by features that are easily observable in a clinical setting.

There are, of course, limitations of using trait-based measures of psychiatric conditions. Further research should be done comparing self measures in diagnosed populations and in participants with no diagnosis of any psychiatric condition. Results from the current study cannot be generalised to comparisons of self-cognition for the associated conditions themselves without such further research. We chose trait-based measures to enable a within-subjects design (as many of our research questions involved investigating variance within individuals), but our choice of psychiatric conditions in each category was also limited by the need for comparable measures. BPD and schizophrenia were the optimal choice for conditions defined in terms of self-cognition, but more varied contrast conditions may have been preferable. It is possible that anxiety and depression are less amenable to self differences because they are both sometimes transient conditions, while our other conditions are developmental or lifelong. Future research in diagnosed populations should also consider using cognitive conditions, such as attention deficit hyperactivity disorder, as additional contrasts to self-defined conditions. These might be candidates for conditions that show no relationship with self-cognition at all.

There is a now long history of considering dimensional approaches both within and across mental conditions as opposed to merely relying on traditional categories based on the presence or absence of symptoms [74–79]. More recent frameworks which endorse the move towards dimensional, diagnostically agnostic, research projects include the National Institute of Mental Health’s Research Domain Criteria (RDOC). This is an alternative research program to the traditional ICD and DSM diagnostic classification systems in which multidimensional neuro-cognitive data drives psychopathological research (see Clark, Cuthbert [80]). The RDOC includes self-cognition as a proposed dimension in two of its domains (systems for social processes and sensorimotor systems). Our results using dimensions of self-cognition to predict psychiatric traits support such a proposal. This allows for a more nuanced understanding of the similarities and differences between psychiatric conditions based on psychophysiological evidence alongside clinical observation.

## Conclusion

In this study, we were interested in the structure of self-cognition. Specifically, we wanted to know whether dimensions of self-cognition across the cognitive hierarchy are associated within an individual; and whether or not such measures support a binary division of psychiatric conditions (based on their traits) along the measured dimensions. In summary, data presented here from 288 participants suggests no relationship between low-level, implicit, attentional self-biases in sensitivity and response time to self-stimuli and higher-order, explicit, self-concept clarity and stability. Nor did either measure of implicit self-prioritisation successfully contribute to predictions of psychiatric trait scores. Our results suggest that while traits of all of our studied psychiatric conditions are associated with a poorer quality of self-concept, the strength of the association comes in varying degrees. As such, the relations between psychiatric traits and explicit self-concept should be conceived as a transdiagnostic spectrum rather than a binary. While self-cognition has shown itself to be multi-dimensional and highly complex, at a minimum, using explicit self-concept as one dimension along which psychiatric conditions differ appears a fruitful avenue for future endeavours.

## Data Availability

Data used for statistical analysis presented below is freely available on Figshare (https://doi.org/10.26180/20011142). A previous dataset using an RT Self-Advantage measure which averages across congruent and incongruent stimuli trials is also available on Figshare (https://doi.org/10.26180/14214464).

## Abbreviations

AQ: Autism Spectrum Quotient
BAI: Beck Anxiety Inventory
BDI: Beck Depression Inventory
BPD: Borderline Personality Disorder
BPQ: Borderline Personality Questionnaire
C1:Explicit: The composite variable derived from loadings on the first component of the principal component analysis that depended on only and all of the explicit self-cognition measures.
C2:Implicit: The composite variable derived from loadings on the second component of the principal component analysis that depended on only and all of the implicit self-cognition measures.
DSM: Diagnostic and Statistical Manual of Mental Disorders
ICD: International Statistical Classification of Diseases
NHST: Null-hypothesis significance testing
PCA: Principal component analysis
RDOC: National Institute of Mental Health’s Research Domain Criteria
RT: Reaction time
SCCS: Self Concept and Identity Scale
SCIM: Self Concept and Identity Measure
SPQ: Schizotypal Personality Questionnaire
